# Applicability of a real-time PCR system to verify labelling compliance of nut allergens in chocolates

**DOI:** 10.1186/2045-7022-3-S3-P129

**Published:** 2013-07-25

**Authors:** J Costa, MBPP Oliveira, I Mafra

**Affiliations:** 1 REQUIMTE, Department of Chemical Sciences, Faculty of Pharmacy, University of Porto, Porto, Portugal

## Background

Chocolate is probably one of the most appreciated food products all over the world. However, for allergic individuals to tree nut ingredients, the enjoyment of a simple milk or black chocolate is denied owing to the excessive use of precautionary labelling practiced by the food industry. Thus, it is of utmost importance the development of accurate analytical methodologies to verify labelling compliance and to contribute for a better control of cross-contamination occurrences during food processing. This work intended to demonstrate the applicability of real-time polymerase chain reaction (PCR) to detect and quantify hazelnut in commercial chocolates.

## Methods

For that, model chocolates spiked with hazelnut were prepared ranging from 10% to 0.001% and 25 chocolates were acquired in the market. Prior to this work, a comparative study of DNA extraction methods elected the NucleoSpin Food kit as the best protocol to assess hazelnut DNA from chocolates [1]. Species-specific PCR was optimised to detect trace amounts of hazelnut in chocolates. Confirmation and quantification results were performed by TaqMan real-time PCR targeting *Hsp1* gene [2].

## Results

The optimisation of both PCR systems allowed detecting hazelnut until 0.005% in chocolate. The application of PCR assays to commercial chocolates was effective, indicating several inconsistencies with labelling. It was also possible to trace hazelnut in chocolates that were not labelled as containing hazelnut as an ingredient, but declared “may contain traces of tree nuts”. Other samples revealed to be excessively labelled since hazelnut was not detected. Real-time PCR confirmed the qualitative PCR results and showed its high potentiality for quantitative analysis of hazelnut allergens in a linear range of 0.005-10%.

## Conclusion

The proposed PCR systems constitute reliable tools for the detection and quantification of nuts in chocolates, allowing tracing minute amounts of hazelnut in difficult matrices such as chocolates.

## Disclosure of interest

None declared.
